# Do education, urbanization, and green growth promote life expectancy?

**DOI:** 10.3389/fpubh.2024.1517716

**Published:** 2025-01-15

**Authors:** Haojun Zhang, Yunqiu Zhan, Keqiu Chen

**Affiliations:** ^1^The Center for Studies of Ethnic Minorities in Northwest China, Lanzhou University, Lanzhou, China; ^2^School of Marxism, Chengdu Technological University, Chengdu, China; ^3^Correspondence Department, Liaoning Technical University, Fuxin, China

**Keywords:** education, urbanization, green growth, life expectancy, China

## Abstract

**Introduction:**

Education (EDU) enhances life expectancy (LEF) by improving health literacy and access to healthcare, leading to healthier lifestyles. Urbanization (URB) fosters better healthcare infrastructure and access to essential services, although it must be managed to avoid negative environmental impacts. Green growth (GG) ensures sustainable development, reduces pollution and environmental risks, and contributes to longer, healthier lives. Therefore, this study examines the impact of EDU, URB and GG on LEF in China from 1990 to 2022.

**Methods:**

This study utilizes the unit root, cointegration test, and Auto Regressive distributed lag (ARDL) model, and for robustness analysis, we use the Fully modified ordinary least squares (FMOLS) and Dynamic ordinary least squares (FMOLS) methods.

**Results:**

The results show that education, urbanization and green growth have a positive and significant effect on life expectancy, while C02 emissions negatively affect life expectancy.

**Discussion:**

These findings suggest that more resources should be allocated to public education systems to ensure access to quality education from early childhood through higher education and integrate comprehensive health education into school curricula to raise awareness about healthy lifestyles, nutrition, and disease prevention. Promote intelligent urban planning incorporating green spaces, recreational areas, and safe walkways to encourage physical activity and reduce pollution. The findings significantly contribute to health economics and provide a new avenue of research for the academic community and policymakers.

## Introduction

**Figure 1:**
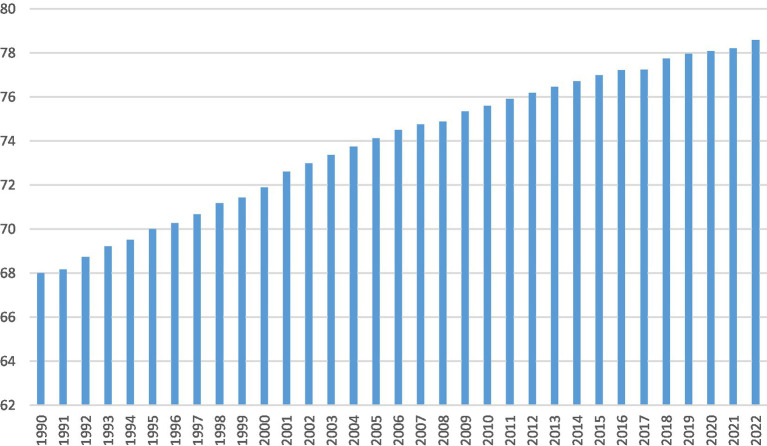
Trends of life expectancy at birth rate in China. Source: WDI.

Health is primarily addressed under SDG 3, which aims to ensure healthy lives and promote well-being for all ages. This goal focuses on reducing maternal and child mortality, combating diseases, and improving healthcare access. Health also supports other SDGs like poverty eradication and quality education by fostering more productive populations. Individuals worldwide require better health facilities, and countries strive to provide their citizens with better health and health-related amenities (1). An essential measure of a country’s health is life expectancy (LEF), which is impacted by social, economic, and environmental factors. Technology, literacy, water, and medical facilities are some of the variables that contribute to the global trend of rising life expectancy (2). The life expectancy at birth estimates how many years a newborn infant would live if the mortality rates at the time of the baby’s birth stayed constant throughout its life (3). Although wealthy countries have improved life expectancy to the desired level, developing countries need help to achieve a fair life expectancy (2). The better modern health system has played a crucial role in the global drop in mortality. However, access to the contemporary health system may differ between rich and poor people (4). Regardless of the state of the economy, all public health systems require consistent financing to maintain essential services such as immunization and screening. In the face of financial constraints, there is also a danger of foregoing cheap preventative care (5, 6). Figure 1 shows the life expectancy trends in China from 1990 to 2022. In 1990, the LEF was approximately 68 years old; it crossed 70 years around 1995, and the milestone of 75 years was reached by 2010. By 2020, it surpassed 78 years, indicating a significant enhancement in public health measures and living conditions. 2020 to 2022 show a gradual increase, with life expectancy rising from 78.072020 to 78.58 in 2022. This reflects ongoing improvements in healthcare and possibly responses to public health challenges. The life expectancy in China has generally increased over the years, rising from 68.005 years in 1990 to 78.58 years in 2022. Over the decades, this indicates improved health, living standards, and medical care.

Educational attainment is a crucial Social determinant of health and a valuable indicator of socioeconomic status (7). The link between increasing education and improved health is well recognized (8, 9). Many studies have found a strong link between education and health outcomes such as life expectancy, healthy aging, cancer, and exposure to risk factors like obesity, substance abuse, and lifestyle factors (10–12). Education directly impacts preventative health by raising awareness of regular health testing, enhancing self-efficacy and confidence, and improving access to health services through increased patience and motivation (13). According to Liu et al. (14) reported that individuals with a greater level of education report better health, maintain a healthier weight, and are less likely to smoke or drink excessively. Parental education has a vital role in reducing child mortality rates; it has been demonstrated that every year of maternal education lowers the risk of under-5 mortality by 3·0%, and every year of paternal education lowers the risk by 1·6% (15, 16). On the contrary, lower education has been linked to increased mortality from all causes, including cardiovascular illnesses, neoplasms, and external causes (17, 18). Several research has linked lower educational attainment or less than high school education to increased all-cause and cardiovascular mortality (7, 19). Health literacy (HL) is one of the most important factors in determining health. Nowadays, HL is a multifaceted idea with a public health viewpoint that characterizes how well people can access, process, and comprehend the fundamental health information they need to make informed decisions about their health (20). Inadequate HL might be a contributing factor to cancer screening inequities and could explain why people are unaware of the significance of getting cancer screening tests (21). Individuals with low HL are more likely to underutilize preventative treatment, increase medical costs by needless hospital stays, and have lower rates of early screenings for diseases like cancer that can be detected early. As a result, these factors increase morbidity and death (22).

Urbanization (URB) refers to human-made settlements with buildings, roads, and infrastructure frequently associated with economic development and improved health due to increased sanitation, education, job opportunities, and access to infrastructure and healthcare facilities. Cities provide more ecological and cultural services, like parks, gardens, and playgrounds. Additionally, sports stadiums and amenities help alleviate stress and promote health (23, 24). Urbanization has increased life expectancy, as city inhabitants earn more and spend more money on their health. The rapid economic advancement and rising urbanization have also expanded access to contemporary, high-tech medical treatment (4). Urbanization has an impact on life expectancy, both positively and negatively. While it frequently leads to increased access to healthcare, education, and higher living standards, unplanned urbanization can also result in environmental degradation, inadequate healthcare infrastructure, and increased exposure to lifestyle-related health risks, potentially lowering life expectancy (2, 25). The rapid and unstructured urbanization process of the 21st century substantially impacts natural and constructed infrastructures, environmental health, and human well-being, emphasizing the role of social dynamics in constructing human society (26–28). Asia and Africa host the world’s largest and fastest-expanding cities. As a result, city living is and will remain the norm for current and future generations globally, with enormous implications for human society, culture, and health (29, 30).

Since environmental deterioration and climate change (CC) have been considered, green development has drawn much attention. Several institutions are taking the green economy seriously, such as the United Nations Economic and Social Commission for Asia and the Pacific (UNESCAP), the World Bank, and the Organization for Economic Cooperation and Development (OECD). The Intergovernmental Panel on Climate Change has given it priority, which is the cause of the ongoing increase in global temperature and its corresponding impact on the globe (31, 32). The Paris Agreement and the 2030 Sustainable Development Agenda have revitalized efforts to improve the environment in order to solve the problems caused by climate change (33). This implies that all developed or developing nations should adhere to Agenda 2030 for Sustainable Development. The nations that prioritize ecologically friendly economic growth differ significantly from one another. Nonetheless, nations with large GDPs are dedicated to preserving the environment. While green growth stagnates or drops in lower-income nations, it significantly increases in high-GDP ones. Consequently, an increase in ongoing economic activity suggests that complex environmental problems cannot be resolved by all available means (34). Green growth (GG) can increase LEF by promoting sustainable practices that reduce environmental risks. Cleaner air and water, resulting from lower emissions and pollution, can improve public health outcomes. Energy-efficient technologies and green industries reduce exposure to harmful chemicals and pollutants.

Moreover, a healthier natural environment fosters active lifestyles and well-being, contributing to longer, healthier lives. The World Health Organization (35) report “Health in the Green Economy: Co-benefits to Health of Climate Change Mitigation.” Household Energy Sector in Developing Countries highlights the health advantages of mitigating climate change by transforming the household energy sector in developing nations. It focuses on reducing reliance on solid fuels (e.g., wood, coal), which currently contribute to air pollution and respiratory diseases. The report argues that shifting to cleaner energy sources—like electricity and modern biofuels—can improve air quality, reduce disease rates, and benefit the environment by lowering greenhouse gas emissions. It recommends policies promoting affordable, clean technologies, which can simultaneously enhance health outcomes and combat climate change.

China was chosen as the study sample because of its alluring carbon neutralization policies for 2060, which is currently the turning point in the global energy transition because of its ecological civilization, technological innovation, environmental policies, and changes in energy production and consumption mechanisms. To accelerate its energy transition, China has adopted several actions. For instance, China has made significant expenditures in its ability to manufacture green energy in the past several years. Consequently, green energy sources provide 50% of the power produced (36). China has progressively expanded healthcare coverage, including establishing universal health insurance schemes like the New Cooperative Medical Scheme (NCMS) for rural areas and the Urban Resident Basic Medical Insurance (URBMI) for urban populations. The “Healthy China 2030” initiative, which aims to create a healthier China over the next 15 years, was implemented by the Chinese government in October 2016. The program’s objectives include ensuring people’s health is maintained throughout their lives, integrating health into all policies, and enhancing health and health equity. Moreover, it will also increase the national LEF to 79 by 2030. Therefore, the following hypotheses are tested in this empirical study:

*H*1: Education has a positive effect on life expectancy.

*H*2: Urbanization has a positive effect on life expectancy.

*H*3: Green growth has a positive effect on life expectancy.

The study contributes to the existing literature in many ways. For instance, it examines the combined impact of EDU, URB, and GG on life expectancy in China from 1990 to 2022. Previous literature regressed green economy indicators such as renewable energy, green technology and green infrastructure on life expectancy but ignored the GG on LEF. This study is the only study that regressed GG on LEF. Therefore, the study’s outcomes provide a better direction for policy implementation. The rest of the study is organized as follows: Section 2 of the literature review sheds light on the previous studies. Section 3 covers the model, methodology and data. Section 4 illustrates the findings and discussion. Section 5 covers the conclusion and policy recommendation.

## Literature review

### Education and life expectancy nexus

Liu et al. (14) investigated the impact of higher education on health in the United Kingdom. They used a quasi-parametric technique for analysis. The result showed that individuals with a greater level of education report better health, maintain a healthier weight, and are less likely to smoke or drink excessively. They suggested that policymakers consider the broader determinants of health and the potential benefits of education in changing health-related behaviors and outcomes.

Iyakaremye and Tripathi (37) examined the impact of education on LEF in Rwanda from 1965 to 2020. They used a vector error correction model (VECM). The findings revealed a significant effect of education on fertility rates, with greater education levels associated with reduced fertility. Furthermore, estimates show an increase in life expectancy across educational levels but less so for the less educated. Gender gaps remain, with male–female life expectancy discrepancies decreasing slower than in prior decades. Increasing prediction intervals indicate increasing uncertainty over time. Balaj et al. (15) conducted a global systematic review and meta-analysis of education’s effects on adult mortality since 1990. The findings indicated that education has a considerable positive effect on reducing all-cause adult mortality, albeit fluctuations in this benefit across time were not statistically significant. Wang et al. (12) used Cox proportional hazard regression to investigate the relationship between education and premature mortality in the Chinese population between 2010 and 2020. They found a strong link between poor education and an increased risk of premature death, with a hazard ratio of 1.93 for individuals with less than elementary education vs. those with higher education levels.

Marlow et al. (38) investigated mortality by education before and during the COVID-19 pandemic in the United States from 2017 to 2020, using data on 7,123,254 deaths, age-standardized-standardized death rates, and mortality rate differences per 100,000 population. Rate ratios comparing the least and most educated were calculated by sex and race/ethnicity. The findings revealed that all-cause mortality rates were around twice as high among those with the most minor education as those with the most education. Disparities in mortality extended dramatically in 2020, with the ratio rising from 2.04 in 2019 to 2.32 in 2020. Notably, unintentional injuries showed the most significant relative rise in mortality inequalities (24.8%).

Addey et al. (39) used Cox regression to investigate the relationship between educational and social inequality and cause-specific death in Mexico City between 1998 and 2004. The findings revealed a considerable inverse relationship between education and premature mortality. Participants with no education had approximately twice the death rate as those with a university education.

Halpern-Manners et al. (40) investigated the effects of education on mortality using linked US Census and administrative mortality data and a twin-difference model. The results revealed strong relationships between education and mortality across all populations, while the estimates were slightly lower among twins and non-twin siblings. This indicates that while education impacts mortality, the effect may be slightly exaggerated without accounting for shared settings. Bijwaard et al. (17) examined the increases in life expectancy related to higher education in men. They analyzed data from the Netherlands using a structural model. The findings revealed significant disparities in life expectancy based on education levels, with higher education being related to longer life expectancy. The study also discusses selection effects, which show that people with greater levels of knowledge have other advantages that help them survive.

The empirical literature examined the EDU-LEF nexus, which reveals consistent evidence of a positive relationship, albeit with variations in magnitude and influencing factors. Studies like Liu et al. (14), Balaj et al. (15), and Halpern-Manners et al. (40) highlight education’s significant role in improving health behaviors, reducing mortality, and increasing LEF across diverse populations. However, differences arise in the degree of impact due to contextual factors. For instance, Iyakaremye and Tripathi (37) emphasize the role of education in reducing fertility and gender gaps in Rwanda, while Marlow et al. (38) and Addey et al. (39) show that the disparities in mortality by EDU are amplified during crises, such as the COVID-19 pandemic. Wang et al. (12) and Bijwaard et al. (17) underscore the heightened risks of premature mortality and reduced LEF among the less educated, often mediated by socioeconomic inequalities. By synthesizing these findings, this study aims to provide a more contextualized understanding of how education shapes life expectancy in China.

### Urbanization and life expectancy nexus

Amin et al. (41) used fully modified ordinary least squares to investigate the effects of urbanization and economic growth on life expectancy in ASEAN-5 nations from 1995 to 2020. Life expectancy is the dependent variable, and the independent variables are health expenditure, economic growth, urban population, and CO2 emissions. Their finding of the detrimental impact of urbanization highlights the possible health risks connected with rapid urban development, necessitating careful urban planning. They suggested strategic urban planning to reduce the health risks associated with urbanization, such as investments in healthcare facilities, sanitation, and public health awareness campaigns. Ahmad et al. (1) used a random effect model to examine the impact of URB and income disparity on male and female LEF in South Asian nations between 1997 and 2021. The results demonstrated that urbanization, wealth disparity, and health expenditure significantly impact life expectancy in both males and females. Urbanization and income inequality reduce life expectancy in both circumstances, although health expenditure increases it. Tripathi (4) conducted a state-level analysis of how cities enhanced our health status in India between 1991 and 2011 utilizing static panel data models, specifically fixed-effect and random-effect models. They found that urbanization positively affects life expectancy at birth, with a 10% increase in urbanization resulting in a 3.3% rise in life expectancy. Kadakia and Galea (29) investigated the link between URB and the future of population health. The results revealed that urbanization has resulted in an “urban health advantage” in many locations, with enhanced health services and sanitation contributing to better health outcomes. Michel (27) investigated the relationship between urbanization and aging health outcomes. They discovered a link between urbanization and health outcomes, demonstrating that metropolitan surroundings can have beneficial and harmful effects on the health of older persons. Jiang et al. (42) investigated the impact of urbanization on population health in China from 2007 to 2019 using a threshold regression model. The findings revealed a strong negative association between urbanization and death rates when the logarithm of per capita GDP is less than a threshold value of 10.237. Beyond this threshold, urbanization’s health-promoting effects decline.

Torres et al. (43) investigated the impact of URB on variations in LEF in Scotland from 1861 to 1910. They used a new decomposition method that divides changes in life expectancy into two major components: changes in mortality and population composition. The findings indicated the occurrence of an “urban penalty,” in which urban regions have higher death rates. Furthermore, an “urbanization penalty” is established, indicating the negative impact of population movement from rural to urban areas on overall survival rates. Ali and Audi (2) used ARDL to examine the association between life expectancy, urbanization, and economic misery in MENA countries between 2001 and 2016. They revealed that urbanization has a considerable and beneficial impact on LEF.

The empirical literature on URB and LEF revealed positive and negative impacts. Studies like Amin et al. (41), Ahmad et al. (1), and Torres et al. (43) highlight adverse effects due to health risks, income inequality, and inadequate planning, while Tripathi (4), Kadakia and Galea (29), and Ali and Audi (2) emphasize positive outcomes such as improved infrastructure and urban health advantages. Jiang et al. (42) reveal a threshold effect, where urbanization’s health benefits diminish beyond certain economic levels. These differences often arise from variations in geography, periods, and socio-economic conditions. This study contributes by making these findings and providing an in-depth understanding of how URB affects LEF in China.

### Green growth and life expectancy nexus

Very limited literature is available on the nexus between green growth and LEF; researchers mostly examine the association between green growth indicators and LEF. Such as Karimi Alavijeh et al. (44) analyzed the nexus between renewable energy (RE) and LEF in G-7 economies from 2000 to 2019. They applied the method of moment-quantile regression (MMQR). The findings showed that while increasing carbon dioxide emissions lowers LEF across all quantiles (5th to 95th), renewable energy, health spending, and urbanization enhance life expectancy across all quantiles (5th to 95th). Mihoub et al. (45) examined the relationship between green energy, sustainable development, and the health system in Saudi Arabia from 1990 to 2022 using machine learning. Overall, the findings of machine learning models indicate a strong impact of digital connectivity on health spending by internet users, with scores of 0.673 and 0.86.

Further, economic growth also influences health costs but to a lesser extent, with scores of 0.145 and 0.082. Mobile user penetration and CO2e have moderate to low importance, suggesting nuanced interactions with health expenditure. Patent applications and logistics performance show minimal impact, indicating a limited direct influence on health costs within this study. Similarly, the share of renewable energy is negligible, reflecting its minimal impact on the analyzed data. Finally, regression analyses using ridge and lasso models confirmed similar trends, further validating these findings. Jiang et al. (23) looked at how green technology and digitalization have affected the health of the BRICS nations between 1993 and 2019. The study empirically investigated country-specific analysis using the ARDL estimation technique. They found that, except in Brazil, digitalization has raised life expectancy over the long term in the BRICS countries. In China and Russia, green technology tends to increase life expectancy over the long term, but its short-term effects on health outcomes are negligible. In the short and long term, life expectancy increases in most BRICS nations due to GDP and health spending. Zhou et al. (46) examined the association between public health events and green economy efficiency in 30 Chinese provinces from 2011 to 2019 by utilizing the four-stage SBM-DEA model and a panel model to construct green economic efficiency indicators. First, it is discovered that public health events significantly impair the effectiveness of the green economy. Second, the influence of public health events on green economic efficiency is significantly moderated by environmental legislation. Third, when environmental regulations become more stringent, the effect of public health events on green economic efficiency shifts from impeding to promoting it. Bowen and Lynch (47) analyzed the public health benefits of green infrastructure. They endorse the effectiveness of using green infrastructure as a climate change adaptation tactic. Green infrastructure may enhance water management, control climate change, and lower air pollution.

Most studies have analyzed EDU, URB, or green growth independently or in limited combinations. There is little research that comprehensively examines their joint impact on LEF, particularly in China, where rapid URB, educational advancements, and green growth policies are intertwined. While some studies, like Zhou et al. (46) and Jiang et al. (23), focus on China, they primarily address green growth and its efficiency or the effects of digitalization and green technology. These studies do not explore how green growth interacts with education and urbanization to shape life expectancy. Therefore, this study fills the gap in the existing literature to examine the impact of education, urbanization, and GG on life expectancy in China.

## Methodology

### Empirical model

The main purpose of this study to analyze the impact of education, urbanization and GG on life expectancy in China from 1990 to 2022. The model use in this study emerged from the previous literature.


(1) $${LEF}_{t}=\emptyset_{0}+\emptyset_{1}{EDU}_{t}+\emptyset_{2}{URB}_{t}+\emptyset_{3}{GG}_{t}+\emptyset_{4}CO2e_{t}+e_{t}$$

In Equation 1, LEF, EDU, URB, GG, and CO2e denote the life expectancy, education, urbanization, green growth, and CO2 emissions, respectively. Where *t* represents a time series data from (1990 to 2022), LEF is dependent variable, and independent variables are EDU, URB, GG, and CO2e. We add CO2e as a control variable. If EDU and GG role plays in the functioning of the rising the LEF, $\emptyset_{1}$ and $\emptyset_{3}$ will be positive. Regarding the empirical literature, URB is positive or negative, $\emptyset_{2}$ will be positive or negative. It is expected that CO2e will be negative effect on LEF, $\emptyset_{4}$ will be negative.

### Estimation technique

In this study, we first test the unit root. Testing for a unit root is important because it helps determine whether a time series is stationary or non-stationary. Non-stationary data with a unit root can lead to spurious regression results, where relationships between variables may appear significant even when they are not. In this we employs the Augmented Dickey-Fuller (ADF), and Phillips-Perron (PP) unit root test. The ADF test developed by Dickey and Fuller (48) and PP test is developed by Phillips and Perron, (49). Equation 2 serves as the foundation for the ADF test.


(2) $$\Delta Z_{t}=\propto ++\beta t+\gamma Z_{t-1}+\overset{p}{\underset{i=1}{\sum }}\delta_{i}\Delta Z_{t-i}+\varepsilon_{t}$$

Where in Equation 2, $\Delta Z_{t}$ is the first difference, ∝ is a drift term, βt is a deterministic time trend, $\gamma Z_{t-1}$ represents the lagged level of the series,$\overset{p}{\underset{i=1}{\sum }}\delta_{i}\Delta Z_{t-i}$ includes the lagged differences of the dependent variable, $\varepsilon_{t}$ is the white noise error term, the super script *p* is the number of lagged difference terms. The null hypothesis (H0): γ=0 implies that there is a unit root (non-stationary series), while the alternative hypothesis (H1): γ<0 suggests stationarity (50). The key feature of the PP test is that it uses non-parametric methods to correct the t-statistic of the γ coefficient for serial correlation and Heteroscedasticity in the residuals without adding lagged difference terms like in the ADF test.

After the unit root test, we employs the cointegation technique such as (JJ) Johansen and Juselius, (51) cointegation test. Testing the cointegration is important because it helps to identify long-run relationships between non-stationary time series variables. If variables are cointegrated, it means they share a common trend and move together over time, even if individually they are non-stationary. Without testing for cointegration, there is risk obtaining spurious results in regression analysis. Co-integration tests, like the JJ cointegation and ARDL bounds tests, ensure that models capture meaningful long-term relationships. Co-integrated variables can be modeled together, improving the accuracy of predictions and policy implications. The JJ approach is ideal for multivariate time series to detect long-run equilibrium relationships. After the cointegration, we thirdly employs the Autoregressive Distributed Lag (ARDL) model, it estimates the both short-run and long-run relationships between variables, in particularly useful when the variables are of mixed order of integration, i.e., when some variables are I(0) or I(1). The ARDL model can be specified as in Equation 3:


(3) $$\begin{array}{l} \Delta LEF_{t}=\beta_{0}+\phi_{1}LEF_{t-1}+\phi_{2}EDU_{t-1}+\phi_{3}URB_{t-1}\\ \;+\phi_{4}GG_{t-1}+\phi_{5}CO2e_{t-1}+\overset{n}{\underset{i=1}{\sum }}\vartheta_{1}\Delta LEF_{t-1}\\ \;+\overset{n}{\underset{i=0}{\sum }}\vartheta_{2}\Delta EDU_{t-1}+\overset{n}{\underset{i=0}{\sum }}\vartheta_{3}\Delta URB_{t-1}\\ \;+\overset{n}{\underset{i=0}{\sum }}\vartheta_{4}\Delta GG_{t-1}+\overset{n}{\underset{i=0}{\sum }}\vartheta_{5}\Delta CO2e_{t-1}+e_{t}\; \end{array}$$

Where difference operator is denoted by delta. The null hypothesis of no long-run relationship existing between the variables ($H_{0}:\phi_{1}=\phi_{2}=\phi_{3}=\phi_{4}=\phi_{5}=0$). If F-value < lower bound, then accept $H_{o}$ and the variables are not co-integrated, if F-value > upper bound, then reject $H_{o}$ and the variables are co-integrated, if, but if F-value ≥ lower bound and ≤ upper bound, then the decision is inconclusive. The (ECM) error correction model for the estimation of the short run relationships are specified as in Equation 4:


(4) $$\begin{array}{l} \Delta LEF_{t}=\beta_{0}+\overset{n}{\underset{i=1}{\sum }}\vartheta_{1}\Delta LEF_{t-1}+\overset{n}{\underset{i=0}{\sum }}\vartheta_{2}\Delta EDU_{t-1}\\ \;+\overset{n}{\underset{i=0}{\sum }}\vartheta_{3}\Delta URB_{t-1}+\overset{n}{\underset{i=0}{\sum }}\vartheta_{4}\Delta GG_{t-1}\\ \;+\overset{n}{\underset{i=0}{\sum }}\vartheta_{5}\Delta CO2e_{t-1}+\forall_{1}ECM_{t-1}+u_{t}\; \end{array}$$

A negative and significant $ECM_{t-1}$ coefficient $\left(\forall_{1}\right)$ implies that any short term disequilibrium between the dependent and explanatory variables will converge back to the long-run equilibrium relationship (52).

After the ARDL estimation, we will test ARDL Diagnostic test, and lastly we further employ the fully modified OLS (FMOLS) and dynamic OLS (DOLS) for the robustness analysis. Both are econometric techniques used to estimate long-run relationships in cointegrated systems. Both methods correct for potential issues like endogeneity and serial correlation that can arise when dealing with non-stationary time series data. FMOLS is a non-parametric correction approach to address serial correlation and endogeneity. While DOLS explicitly adds leads and lags of first differences to deal with the same issues [see, (53, 54); Table 6].

**Table 1:** Variable measurement.

Symbol(s)	Variable(s)	Measurement(s)	Source(s)
LEF	Life expectancy	at birth, total (years)	WDI
EDU	Education	Mean Years of Schooling	UNDP
URB	Urbanization	Urban population (% of total population)	WDI
GG	Green growth	Environmentally adjusted multifactor productivity	OECD
CO2e	Carbon emissions	metric tons per capita	WDI

Source: Authors compilation.

### Data and variables

This study examines the impact of education, urbanization, and green growth on LEF in China from 1990 to 2022. The data has been obtained from the World Development Indicators (WDI), United Nation Development Program (UNDP) and Organization for Economic Cooperation and Development (OECD) websites. Table 1 shows the Variable measurement.

## Results

### Descriptive statistics

Table 2 displays the descriptive statistics, the mean value of LEF, EDU, URB, GG and CO2e are 73.887, 6.366, 44.237, 4.886 and 4.827, respectively. The median value of LEF, EDU, URB, GG and CO2e are 74.504, 6.407, 43.868, 4.474 and 4.910, respectively. The standard deviation of LEF, EDU, URB, GG and CO2e are 3.290, 1.166, 11.731, 1.414 and 2.221, respectively. The null hypothesis (*H_0_*) of the Jarque-Bera test assumes the variable is normally distributed. A higher *p*-value (> 0.10) suggests failure to reject *H_0_* (i.e., the variable is likely normal). A low *p*-value (≤ 0.10) indicates a departure from normality. The variables LEF, EDU, URB and CO2e are normally distributed except, GG.

**Table 2:** Descriptive statistics.

	LEF	EDU	URB	GG	CO2e
Mean	73.887	6.366	44.237	4.886	4.827
Median	74.504	6.407	43.868	4.474	4.910
Maximum	78.587	8.107	63.560	11.185	7.756
Minimum	68.005	4.144	26.442	3.007	1.915
Std. Dev.	3.290	1.166	11.731	1.414	2.221
Skewness	−0.302	−0.206	0.089	3.020	0.027
Kurtosis	1.809	2.084	1.690	13.531	1.288
Jarque-Bera	2.453	1.387	2.405	202.662	4.035
Probability	0.293	0.500	0.300	0.000	0.133

### Unit root and cointegration test results

Table 3 presents the outcomes of the ADF and PP unit root test. The outcomes of ADF reported that with constant LEF and GG are stationary at level, URB and GG are stationary at level with constant & trend, while EDU and CO2e are non-stationary at level. After the first difference all variables becomes stationary. The results of PP reported that LEF and GG are stationary at level, while all variables becomes stationary after first difference. So we concluded that there are mixed order of the data stationary. So we will used the ARDL methods. Table 4, shows the results of Johansen cointegration and ARDL bound test. The Johansen cointegration test, Trace statistics confirms the 4 cointegated equation, while Max-Eigen statistics confirms the 3 cointegated equation. Table 4 also shows the assessed value of the ARDL bound test, the F statistic is greater than the 8.560 > 5.53, so the data confirms that there is cointegration among the variables.

**Table 3:** Unit root test.

At Level						
		LEF	EDU	URB	GG	CO2e
ADF test						
With Constant	t-Statistic	−2.803^***^	−1.707	−1.067	−3.305^**^	−0.846
	Prob.	0.069	0.418	0.715	0.023	0.792
With Constant & Trend	t-Statistic	−0.416	−2.244	−3.369^***^	−3.257^***^	−1.809
	Prob.	0.983	0.450	0.074	0.092	0.676
First Difference						
With Constant	t-Statistic	−5.118^*^	−1.213	−4.793^*^	−6.044^*^	−2.747^***^
	Prob.	0.000	0.656	0.000	0.000	0.078
With Constant & Trend	t-Statistic	−5.529^*^	−3.751^*^	−0.898	−5.942^*^	−2.721
	Prob.	0.001	0.001	0.943	0.000	0.236
PP test						
With Constant	t-Statistic	−3.143^**^	−2.504	1.468	−2.942^***^	−0.567
	Prob.	0.033	0.124	0.999	0.052	0.864
With Constant & Trend	t-Statistic	−0.239	−2.056	−2.846	−2.847	−1.463
	Prob.	0.989	0.550	0.192	0.192	0.821
First Difference						
With Constant	t-Statistic	−5.281^*^	−1.213	−4.690^*^	−11.317^*^	−2.712^***^
	Prob.	0.000	0.656	0.000	0.000	0.083
With Constant & Trend	t-Statistic	−7.419^*^	−3.851^*^	−0.330	−11.442	−2.675
	Prob.	0.000	0.000	0.986	0.000	0.253

*, ** & *** indicates the significance level at 1, 5% & 10%.

**Table 4:** Cointegration test.

Johansen cointegration					
Hypothesized	Trace			Max-Eigen	
No. of CE(s)	Eigenvalue	Statistic	Prob.	Statistic	Prob.
None	0.602^*^	78.841	0.008	68.581^**^	0.009
At most 1	0.494^**^	50.260	0.029	41.124^**^	0.056
At most 2	0.336^***^	29.136	0.060	22.678^***^	0.084
At most 3	0.287^**^	16.457	0.036	15.504^*^	0.099
At most 4	0.175^**^	5.954	0.015	1.954	0.515
ARDL bound test					
Test statistic	Value	Critical value			
F-statistic K	8.560^*^ 4	Significance	I (0)	I (1)	
		10% 5% 1%	2.460 2.947 4.093	3.460 4.088 5.532	

*, ** & *** indicates the significance level at 1, 5% & 10%.

### ARDL estimation results

Table 5 illustrates the results of the ARDL test. In the long run, the coefficient of EDU is positive, indicating that a 1% increase in EDU will surge the LEF by 0.151% at a 1% significance level in China from 1990 to 2022. It rejects the null hypothesis of no nexus between EDU and LEF.

**Table 5:** ARDL estimates.

Long run				Short run		
Variable	Coefficient	SE	Prob.	Coefficient	SE	Prob.
EDU	0.151^*^	0.052	0.004	0.946^*^	0.350	0.013
URB	0.468^*^	0.041	0.000	1.229^*^	0.139	0.000
GG	0.320^*^	0.020	0.000	0.003	0.011	0.781
CO2E	−0.482^*^	0.099	0.000	−0.510^*^	0.095	0.000
C	58.719^*^	13.461	0.000			
ECM(−1)				−0.887^*^	0.112	0.000
ARDL Diagnostic analysis						
Test			F stats	Prob.		
Normality test-Jarque–Bera			0.034	0.984		
Autocorrelation test-LM t			1.161	0.452		
Heteroscedasticity test-ARCH			0.772	0.631		
Stability test-Ramsey RESET			1.252	0.242		

*, ** & *** indicates the significance level at 1, 5% & 10%. Selected Model: ARDL (1, 1, 1, 1, 1). Dependent variable LEF.

The coefficient of URB is positive, showing that a 1% increase in urbanization leads to a surge in LEF by 0.468%. The coefficient of GG is positive, showing that a 1% increase in green growth leads to a surge in LEF by 0.320%. The coefficient of CO2e is −0.482, showing that a 1% increase in CO2e leads to reduced LEF by 0.482%. Table 5 also shows the short-run ARDL estimates. EDU, URB, and GG positively affect LEF, while CO2e negatively affects LEF China from 1990 to 2022. The error correction mechanism (ECM) coefficient is negative −0.887 and significant. It indicates the 88.7% rate at which the dependent variable returns to equilibrium following a deviation.

### ARDL diagnostic and robustness analysis results

**Figure 2:**
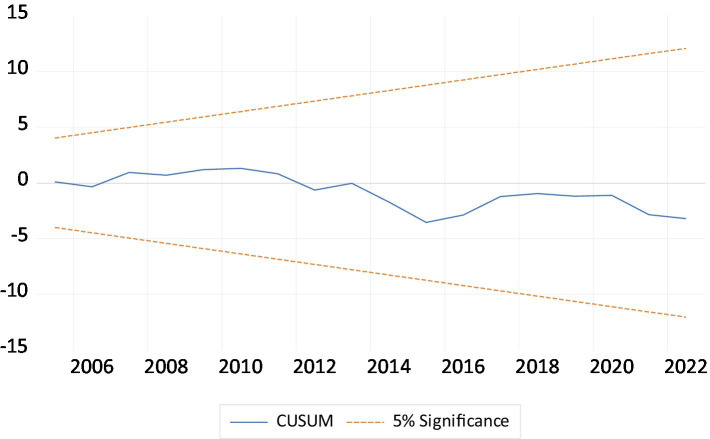
The cumulative sum of the recursive residual plot.

**Figure 3:**
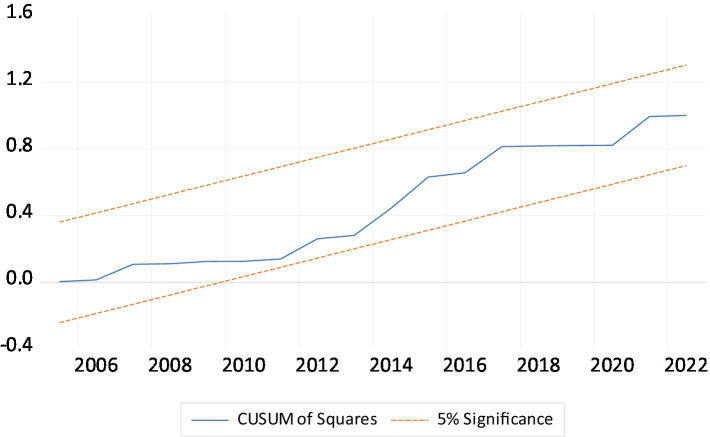
The cumulative sum of the square of the recursive residual plot.

As shown in Table 5, ARDL diagnostic analyses examine the LEF models. Diagnostic tests indicated that Autocorrelation and Heteroscedasticity were not issues in the ARDL model. The Ramsey RESET and the Jarque–Bera statistic accepted the stable model and normal distribution of residual of the ARDL model. The CUSUM (Cumulative Sum) and CUSUMsq (Cumulative Sum of Squares) tests are shows in Figures 2, 3. Both tests are diagnostic tools used to assess the stability of model parameters over time, particularly in time series analysis, such as in ARDL models. The reliability of policy replication based on the sample period outcomes depends on parameter stability. Since the red line falls within the critical boundaries (represented by blue lines at the 5% significance level), Figures 2, 3 indicate that the LEF models (both short-run and long-run) are well-specified. This demonstrates that the model parameters are robust and produce consistent results. For the robustness analysis we used the FMOLS and DOLS estimators for time series data also used by Azam et al. (55), Khan et al. (56) and Khan et al. (57). The robustness analysis of both FMOLS and DOLS tests confirms the same finding regarding the ARDL long-run estimates, but their magnitude and significance levels differ. According to the findings, the EDU, URB, and GG have a positive effect on LEF, while CO2e has a negative effect on LEF (Table 6).

**Table 6:** Robustness analysis.

FMOLS				DOLS		
Variable	Coefficient	SE	Prob.	Coefficient	SE	Prob.
EDU	2.692^*^	0.287	0.000	1.671^*^	0.146	0.000
URB	0.101^**^	0.043	0.026	0.241^*^	0.024	0.000
GG	0.055^***^	0.028	0.062	0.095^*^	0.011	0.000
CO2e	−0.604^*^	0.106	0.000	−0.364^*^	0.063	0.000
C	58.048^*^	0.515	0.000	57.960^*^	0.372	0.000
Adj. R^2^	0.982			0.996		

*, ** & *** indicates the significance level at 1, 5% & 10%. Dependent variable LEF.

## Discussion

In the long run, the coefficient of Education is positive effect on LEF. Education increases LEF by promoting healthier lifestyles, better access to healthcare, and improved health literacy. Educated individuals are more likely to understand health risks, adopt preventative measures, and make informed diet, exercise, and medical care decisions. Moreover, EDU can lead to better economic opportunities, reducing poverty-related health risks and improving access to clean water, sanitation, and nutritious food. The results are consistent with the lines of Liu et al. (14), Iyakaremye and Tripathi (37), and Bijwaard et al. (17). Liu et al. (14) showed that individuals with a greater level of education report better health, maintain a healthier weight, and are less likely to smoke or drink excessively. Iyakaremye and Tripathi (37) revealed a significant effect of education on fertility rates, with greater education levels associated with reduced fertility. Furthermore, estimates show an increase in life expectancy across educational levels but less so for the less educated. Bijwaard et al. (17) revealed significant disparities in life expectancy based on education levels, with higher education related to longer life expectancy. The study also discusses selection effects, which show that people with greater levels of knowledge have other advantages that help them survive.

The urbanization is positive effect on LEF, urbanization increases LEF by improving access to healthcare, clean water, and sanitation infrastructure. Urban areas often provide better medical services, disease prevention programs, and emergency care. URB promotes higher education levels, contributing to healthier lifestyles and better health awareness. Moreover, economic opportunities in cities often lead to improved living conditions, nutrition, and overall well-being, helping reduce mortality rates and extend LEF. The finding is consistent with the findings of Tripathi (4) and contradicts Amin et al. (41) and Ahmad et al. (1). Tripathi (4) reported that urbanization has a positive effect on LEF. Amin et al. (41) found that a detrimental impact of urbanization highlights the possible health risks connected with rapid urban development in ASEAN-5 nations, necessitating careful urban planning. Ahmad et al. (1) reported that urbanization and income inequality reduce life expectancy in South Asian countries.

The green growth is also positive effect on LEF, Green growth boosts LEF by promoting sustainable development that reduces environmental pollution and improves public health. Green growth minimizes health risks such as respiratory and cardiovascular diseases by transitioning to cleaner energy, reducing emissions, and enhancing air and water quality. The finding is consistent with Jiang et al. (23) and Karimi Alavijeh et al. (44). Jiang et al. (23) reported that green technology increases life expectancy. Karimi Alavijeh et al. (44) found that renewable energy has raised the LEF.

The CO2e coefficient is negative effect on LEF, the reason of the negative sign in that CO2e reduces LEF by contributing to air pollution, increasing respiratory and cardiovascular disease risk. Long-term exposure to high levels of CO2e and associated pollutants can worsen public health, leading to higher mortality rates. Additionally, climate change driven by CO2e exacerbates extreme weather events, food insecurity, and the spread of diseases, further negatively impacting LEF. The finding is consistent with the findings of Azam et al. (55) and Uddin et al. (58). Both reported that CO2e reduces the LEF in Pakistan and South Asia.

## Conclusion and policy recommendation

This study empirically examined the impact of education, urbanization, and green growth on LEF in China from 1990 to 2022 using the ARDL approach to co-integration analysis. The findings showed that urbanization, education, and green growth have a positive effect on LEF, while CO2e has a negative effect on LEF in China.

This study recommends some suggestions for policymakers for health and social well-being through increased life expectancy in China: first, increase funding for public health facilities, ensuring they are accessible, affordable, and equipped to handle a diverse range of health issues. Second, Promote thoughtful urban planning incorporating green spaces, recreational areas, and safe walkways to encourage physical activity and reduce pollution. Third, stricter industrial emissions and vehicle pollution regulations should be enforced to improve air quality, thereby protecting residents’ respiratory health. Fourth, more resources should be allocated to public education systems to ensure access to quality education from early childhood through higher education. Fifth, Promote programs that provide free or subsidized early childhood education to lay a strong foundation for lifelong learning. Sixth, comprehensive health education should be integrated into school curricula to raise awareness about healthy lifestyles, nutrition, and disease prevention. Seventh, Create initiatives that foster parental involvement in education, as engaged parents can positively influence their children’s academic performance and health. Eight, Support policies that incentivize the transition to renewable energy sources, such as solar, wind, and hydroelectric power, to reduce pollution and greenhouse gas emissions and improve health. Ninth, invest in developing and maintaining urban green spaces, parks, and community gardens to improve public health and encourage outdoor activities.

Lastly, several limitations identified in this study will inform future investigations. Future research will use these variables to assess developed, emerging, and developing nations, as we only considered the Chinese economy. Only we utilized some variables and ignored the numerous macroeconomic, demographic, social, and health variables that influence LEF. We did not employ asymmetric analysis or quintile regression and structural break; future research will be used to extend the current study by using this technique.

## Data Availability

Data used in this study for empirical examination have been obtained from the World Development Indicators, UNDP, and OECD, given in Table 1. Further inquiries can be directed to the corresponding author.
